# Novel mutations in *PLCZ1* lead to early embryonic arrest as a male factor

**DOI:** 10.3389/fcell.2023.1193248

**Published:** 2023-05-16

**Authors:** Yunying Lin, Yi Huang, Boyu Li, Ting Zhang, Yichao Niu, Shuanggang Hu, Ying Ding, Guangxin Yao, Zhe Wei, Ning Yao, Yejie Yao, Yao Lu, Yaqiong He, Qinling Zhu, Ling Zhang, Yun Sun

**Affiliations:** ^1^ Center for Reproductive Medicine Center, Renji Hospital, School of Medicine, Shanghai Jiao Tong University, Shanghai, China; ^2^ Shanghai Key Laboratory for Assisted Reproduction and Reproductive Genetics, Shanghai, China

**Keywords:** male infertility, *PLCZ1* bi-allelic mutation, early embryonic developmental arrest, polyspermy, ICSI with AOA

## Abstract

Early embryonic arrest is one of the causes of assist reproduction technology (ART) failure. We have previously reported that the first sperm-derived genetic factor, *ACTL7a* mutations, could lead to early embryonic arrest. However, whether there are other male genetic factors associated with early embryonic arrest remains elusive. Here, we reported bi-allelic mutations in *PLCZ1*, a well-known causal gene of total fertilization failure, in four infertile males. Among these mutations, p.403_404del, p.I489S, and p.W536X were newly reported in this study. Histological and Western blotting analysis of the patients’ sperm indicated these variants as loss-of-function mutations. These patients manifested normal conventional semen parameters and ultra-structures in sperm heads. However, among four *in vitro* fertilization (IVF) cycles, 81.8% (18/22) of the oocytes were polyspermic fertilized, which was rarely reported in *PLCZ1*-related male patients. In the following six ICSI cycles, artificial oocyte activation (AOA) was applied and successfully rescued the fertilization failure and polyspermy phenotypes, with 31.3% (15/48) of the MII oocytes normally fertilized. However, 60.0% (9/15) of these normally fertilized zygotes were arrested at 2–5-cell stage, with one failing to cleave, indicating that *PLCZ1* was not only necessary for fertilization, but also crucial for early embryonic development. However, these rescued zygotes showed a lower potential in developing into blastocysts when cultured *in vitro*. Thus, fresh cleavage transfer was tried and two live births were successfully achieved thereafter. In conclusion, this study provided novel mutations in *PLCZ1* gene to expand the pathogenic mutational spectrum in male infertility and demonstrated that *PLCZ1* was a crucial sperm-related genetic factor for early embryonic arrest. We also proposed that cleavage transfer after ICSI and AOA treatment could be a potential treatment method for male patients carrying bi-allelic mutations in *PLCZ1*.

## Introduction

It has been widely accepted that phospholipase C zeta (*PLCZ1*), localized in the acrosome in spermatozoa, is a causal factor that leads to fertilization failure (FF) after intracytoplasmic sperm injection (ICSI) due to its ability in inducing the characteristic calcium oscillations to stimulate meiotic progression (Saunders et al., 2002; Kashir et al., 2011; Nomikos et al., 2013; Kashir et al., 2014; Yelumalai et al., 2015; Nikiforaki et al., 2016; Sanders and Swann, 2016; Swann and Lai, 2016; Yeste et al., 2016; Kashir, 2020). It has been reported that one-third of infertile men suffering from FF carry mutations in *PLCZ1* gene (Escoffier et al., 2016; Dai et al., 2020). An increasing number of bi-allelic *PLCZ1* mutations have been reported since its discovery. Nowadays, *PLCZ1* expression level in the sperm is often used as a biomarker for prediction of fertilization potential after ICSI (Kashir et al., 2013; Torra-Massana et al., 2019; Cheung et al., 2020; Kashir et al., 2020). Expanding the mutational spectrum of *PLCZ1* helps to provide theoretical support for more infertile men.

In *Plcz1* ^*−/−*^ animal models, both FF and polyspermy phenotypes were reported (Hachem et al., 2017; Nozawa et al., 2018). FF, characterized as failure to form two-pronucleus (2 PN) zygotes with morphologically normal gametes even with the help of ICSI, was a typical phenotype of infertile men carrying bi-allelic mutations in *PLCZ1* (Mahutte and Arici, 2003; Torra-Massana et al., 2019; Yan et al., 2020). Polyspermy is defined as fertilization of an oocyte by more than one sperm (Evans, 2020). However, the polyspermy phenotype was most recently reported in only one study, which presented an infertile male with a homozygous *PLCZ1* mutation (Peng et al., 2023). More cases are needed to further advocate the causal relationship between *PLCZ1* bi-allelic mutations and the polyspermy phenotype.

Mutations in one gene may lead to various phenotypes. People carrying variants in *ACTL7* displayed not only fertilization failure but also early embryonic arrest (Xin et al., 2020; Wang et al., 2021). According to previous study, disruption in ACLT7A protein could lead to embryonic arrest at 2-5-cell stage in mice and PLCZ1 protein deletion was the crucial result of *Aclt7a* deficiency. Although a delay in development was found in embryos fertilized with sperms from *Plcz*1^−/−^ mice (Wang et al., 2022), whether *PLCZ1* deficiency was associated with early embryonic arrest in human remained further study.

According to previous studies, artificial oocyte activation (AOA) could rescue the lack of Ca^2+^ oscillations caused by mutations in *PLCZ1*, thus rescuing the oocytes from fertilization failure and increasing the 2PN rate (Dai et al., 2020; Peng et al., 2023). However, only one fourth of the patients with bi-allelic *PLCZ1* mutations treated by ICSI with AOA could have their own babies (Mu et al., 2020; Yan et al., 2020), indicating that *PLCZ1* played an important role not only in fertilization, but also in embryonic development.

In this study, among four *PLCZ1*-related infertile male patients, we identified three novel pathogenic mutations, which expanded the mutational spectrums of *PLCZ1* that caused male infertility. Besides, more cases were reported to further verify the polyspermy phenotype in *PLCZ1*-related infertility. Moreover, early embryonic arrest was identified as a new phenotype caused by bi-allelic mutations in *PLCZ1*, which further proved that the male factor could lead to early embryonic arrest. Finally, based on the fact that mutations in *PLCZ1* affected the early embryonic development, an attempt of fresh cleavage transfer was made for our patient and two live births were achieved, which provided a possible treatment for male patients with bi-allelic *PLCZ1* mutations in the future.

## Materials and methods

### Clinical samples

The clinical samples consisted of a total of 60 infertile Chinese couples who exhibited fertilization disorder upon multiple IVF and ICSI cycles (Supplementary Figure S1). All the individuals were recruited from Reproductive Center of Renji Hospital affiliated to Shanghai Jiao Tong University School of Medicine. The ethics were approved by the ethics committee of Renji Hospital. All patients and their parents were given written informed consent before the study.

### Semen analysis and sperm preparation

After 3–7 days of sexual abstinence, semen samples were collected by masturbation and were examined after liquefaction for 30 min at 37°C. In clinical practice, the semen parameters were analyzed in terms of the fifth edition of the WHO laboratory manual. Multiple indexes of the semen samples were assessed with the light microscope. The normal semen should present >15 × 10^6^/mL concentration, 40% total motility (>32% progressive motility), ≥4% morphologically normal sperm rate, ≤1 × 10^6^/mL round cell concentration, and ≤15% sperm DNA fragmentation rate. At least two biological replication were prepared and analyzed for the semen analysis. For evaluation of sperm morphology, 20 μL of semen was spread on the slides, dried at room temperature, and fixed in 95% ethanol for Papanicolaou stain. Spermatozoa were then assessed by ×100 oil-immersion bright-field objective. At least 200 spermatozoa were examined.

### Whole-exome sequencing and bioinformatic analysis

Genomic DNAs were extracted from 2 mL peripheral blood from all participants and their available parents by following the instructions of the AllPrep DNA/RNA/Protein Mini Kit (QIAGEN, Germany). Whole-exome sequencing (WES) was performed using the Agilent SureSelect Whole Exome capture and paired-end sequencing on Illumina sequencing platform following the standard procedures. The reads were aligned to the human genome reference assembly (hg19) with the Burrows-Wheeler Aligner. The candidate variants met the following criteria: 1) homozygous or compound heterozygous missense, nonsense, splicing site, and indel variants; 2) variants with a minor allele frequency <0.1% in the public human genome databases of the 1000 Genomes Project, the ExAC Browser and the gnomAD; 3) variants located within homozygous regions greater than 2.0 Mb; 4) variants functionally predicted by at least one prediction software to be deteriorating. SIFT, Mutation Taster and PolyPhen-2 were used as predictors for deleterious variants.

### Sanger sequencing

Sanger sequencing was used to confirm the candidate variations in all available members of the families. The primers used to amplify *PLCZ1* mutations were shown in Supplementary Table S1. PCR amplification were carried out as follows: denaturation at 98°C for 1 min, followed by 35 cycles of amplification (98°C for 10 s, 60°C for 15 s and 72°C for 45 s) and an elongation step at 72°C for 5 min. Sequence analyses were carried out using the ABI 3730XL (Applied Biosystems, United States).

### Molecular modeling

The wild-type and the variant models of PLCZ1 protein 3D structure were generated based on the predicted result of Phyre2 database (http://www.sbg.bio.ic.ac.uk/phyre2), and were mapped onto the atomic model using PyMol software.

### Transmission electron microscopy (TEM)

The washed human sperm samples from the affected individuals identified in our study were fixed in 2.5% glutaraldehyde for 24 h at 4°C to investigate sperm ultrastructures. The specimens were embedded in Epon 812, cut into 70- to 90-nm-thick ultrathin sections, and were then stained with uranyl acetate and lead citrate for subsequent observation and photography by TEM (Tecnai-10, Philips, Netherlands).

### Immunofluorescence staining

The washed sperm samples from the affected individuals identified in our study and from the control donor were fixed with 4% paraformaldehyde (Sangon Biotech, China) for 1 h at room temperature, followed by two washes with PBS, and then were smeared onto polylysine-coated slides. After drying, sperm samples were then subjected to permeabilization with 1% Triton X-100 (Sigma, United States) and blocked with 10% donkey serum albumin (Jackson ImmunoResearch, United States) for 1 h at room temperature. The slides were then incubated with rabbit polyclonal anti-PLCZ1 antibody (1:100; Invitrogen, United States) and alpha Tubulin mouse monoclonal antibody (B-5-1-2), Alexa Fluor™ 488 (1:500; Abcam, UK) overnight at 4°C. After being washed for 3 times with PBST, the slides were incubated with Alexa Fluor™ 647 goat anti-rabbit immunoglobulin G (IgG) secondary antibody (1:500; Invitrogen, United States) for 1 h at room temperature. Finally, the sections were mounted with one drop of DAPI (4′,6-diamidino-2-phenylindole) Fluoromount-G (SouthernBiotech, United States) to label the DNA for image acquisition using the Nikon A1 + Confocal Microscope System (Japan).

### Western blotting analysis

Ejaculated human semen was obtained from patients or from control donors with fertilization rate over 50%. The precipitates were collected from the washed semen samples following centrifugation (3,000 rpm for 3 min) and lysed in RIPA buffer (Beyotime, China) containing protease inhibitors cocktail (Roche Diagnostics, Germany) for 30 min at 4°C. The samples were then centrifuged at 12,000 × g for 30 min at 4°C. Supernatants were collected, mixed with 5× sodium dodecyl sulphate (SDS) loading buffer, and heated at 100°C for 5 min for subsequent Western blotting analysis. The protein samples were subjected to electrophoresis using 10% SDS polyacrylamide gels and transferred to nitrocellulose membranes. Bands with peroxidase activity were detected using a chemiluminescent detection kit (MilliporeSigma, United States) and visualised with a G-Box chemiluminescence image capture system (Syngene, Frederick, United States). The relative abundance of a target protein to that of intensity of GAPDH was analysed using Gelpro software and obtained as each target protein level. The following primary antibodies were used: anti-PLCZ1 antibody (1:1000; Invitrogen, United States); anti- GAPDH antibody (1:20000; Proteintech, China).

## Results

### Novel pathogenic mutations were identified in *PLCZ1* from infertile males

**FIGURE 1:**
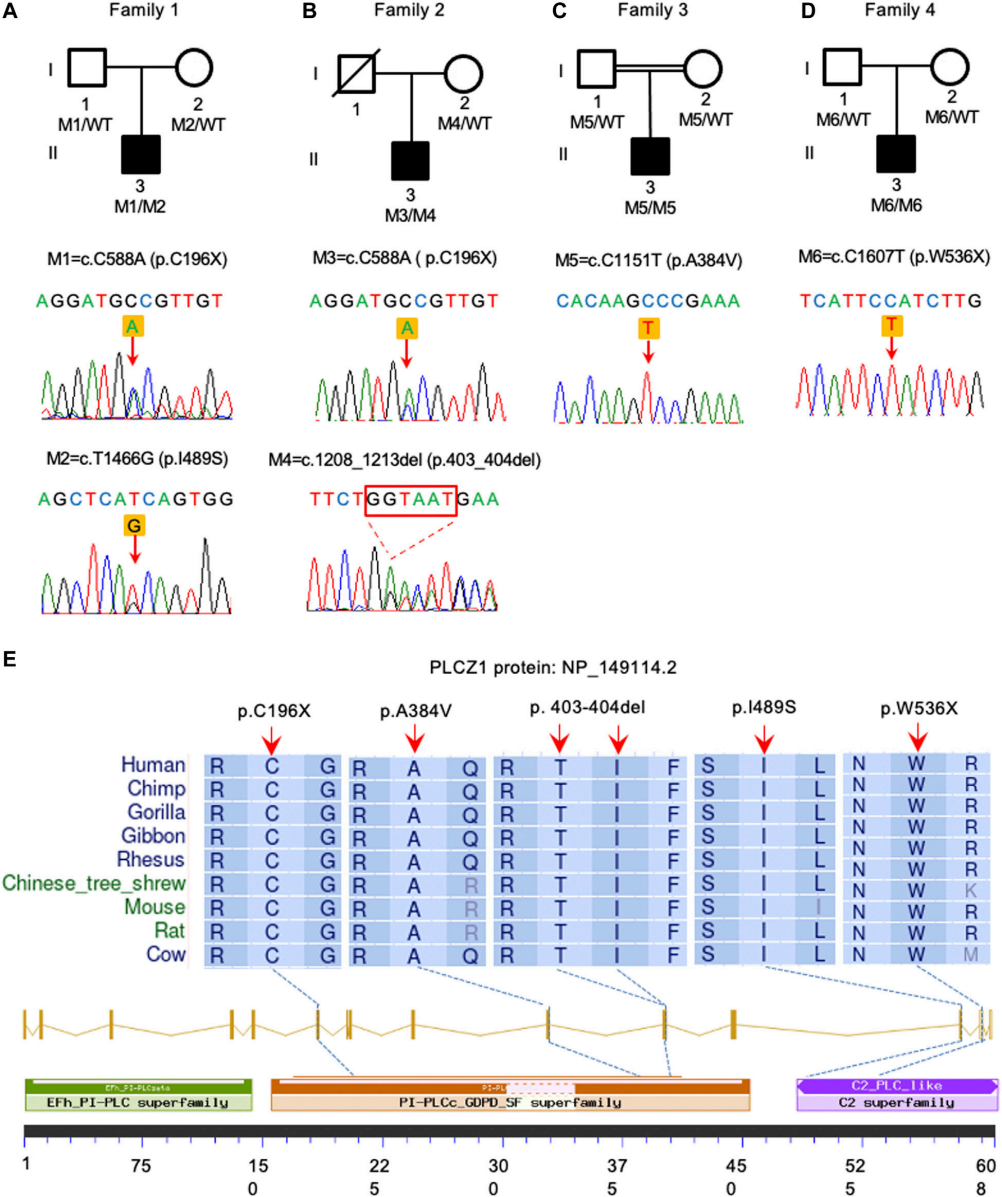
Identification of *PLCZ1* bi-allelic mutations in infertile males. **(A–D)** Pedigrees of four families affected by *PLCZ1* bi-allelic mutations. Sequences of mutations were shown below. Black squares indicate the male individuals with *PLCZ1* mutations. **(E)** Variant locations and phylogenic conservation of the affected residuals in the PLCZ1 protein. The NCBI reference number for PLCZ1 protein is NP_149114.2.

In this study, 60 genetically independent infertile male patients suffering from fertilization disorder were collected for WES analysis. We identified compound heterozygous mutations of (p.C196X, p.403_404del) and homozygous mutation of p.W536X in *PLCZ1* gene in two patients with fertilization failure. Compound heterozygous mutations of (p.C196X, p.I489S) and homozygous mutation of p.A384V were identified in two patients with polyspermy. Notably, p.I489S, p.403_404del and p.W536X were novel mutations reported in this study (Figures 1A–D; Table 1).

**TABLE 1:** Bi-allelic *PLCZ1* variants identified in the patients.

	Patient 1		Patient 2		Patient 3	Patient 4
cDNA alteration	c.C588A	c.T1466G^a^	c.C588A	c.1208_1213del^a^	c.C1151T	c.C1607T^a^
Variant allele	Het	Het	Het	Het	Hom	Hom
Protein alteration	p.C196X	p.I489S	p.C196X	p.403_404del	p.A384V	p.W536X
Variant type	Stop gain	Missense	Stop gain	Inframe deletion	Missense	Stop gain
Allele frequency in human population						
1000 Genomes	0.0002	0	0.0002	0	0	0
gnomAD	0.00001	0	0.00002	0	0.000004	0
Function prediction						
SIFT	NA	D	NA	NA	D	NA
PolyPhen-2	NA	D	NA	NA	D	NA
MutationTaster	A	D	A	NA	D	A

NCBI, reference sequence number of *PLCZ1* is NM_033123.4.

A, disease causing; D, damage; Het, heterozygous; Hom, homozygous; NA, not applicable.

^a^ Novel mutations reported in this study.

All mutations were conserved among species in different domains of *PLCZ1*, including one (p.C196X) in X domain, two (p.A384V and p.403_404del) in Y domain, and two (p.I489S and p.W536X) in C2 domain (Figure 1E). Although no evidence showed racial differences in this gene in previous studies, the carrier frequency of reported and expected *PLCZ1* mutations that causes disease in East Asian population is much higher than that in the overall population (0.00123 vs. 0.00081) according to the gnomAD database, especially for mutations in the X domain (0.00076 vs. 0.00006) and Y domain (0.00076 vs. 0.00007) (Table 2). This result suggested a higher risk of bi-allelic mutations in *PLCZ1* causing male infertility in East Asian population (Table 2).

**TABLE 2:** Analysis of the domain and allele frequency of the novel and reported mutations.

Mutation site	Domain	Mutation type	LOF	East Asian allele frequency (gnomAD)	Total allele frequency (gnomAD)	References
c.1733T>C (p.M578T)		Missense		0.0002518	0.00001777	Yan et al. (2020)
						Yuan et al. (2020b)
c.1727T>C (p.L576P)		Missense		NA	NA	Yuan et al. (2020b)
c.1658 G>C (p. R553P)	C2	Missense		NA	NA	Yuan et al. (2020a)
c.1607C>T (p.W536X)	C2	Stop gain	√	NA	NA	Novel
c.1466T>G (p.I489S)	C2	Missense		NA	NA	Novel
c.1465A>T (p.I489F)	C2	Missense		0	0.000004031	Escoffier et al. (2016)
**Total in C2 domain**				**0**	**0.000004031**	
c.1358G>A (p.G453D)	Y	Missense		0	0.000003985	Zhao et al. (2023)
c.1344A>T (p.K448N)	Y	Missense		NA	NA	Yan et al. (2020)
c.1274A>G (p.N425S)	Y	Missense		NA	NA	Zhao et al. (2023)
c.1259C>T (p.P420L)	Y	Missense		NA	NA	Yuan et al. (2020b)
						Mu et al. (2020)
c.1234delA (p.Arg412fs)	Y	Frameshift deletion	√	0.0001538	0.00001094	Mu et al. (2020)
c.1208_1213del (p.403_404del)	Y	In frame deletion		NA	NA	Novel
c. p.H398P	Y	Missense		0	0.000007356	Kashir et al. (2012)
c.1174+3A>C		Splicing		NA	NA	Zhao et al. (2023)
c.1151C>T (p.A384V)	Y	Missense		0.00005441	0.000003986	Yan et al. (2020)
c.1129_1131delAAT (p.N377del)	Y	In frame deletion		0.0005517	0.000039	Yan et al. (2020)
c.1048T>C (p.S350P)	Y	Missense		NA	NA	Kashir et al. (2012)
**Total in Y domain**				**0.00075991**	**0.000065267**	
c.972_973delAG (p.T324fs)	XY linker	Frameshift deletion	√	NA	NA	Mu et al. (2020)
c.830T>C (p.L277P)	X	Missense		NA	NA	Yan et al. (2020)
c.736C>T (p.L246F)	X	Missense		NA	NA	Kashir et al. (2012)
c.698A>T (p.H233L)	X	Missense		0	0.0006864	Kashir et al. (2012)
c.590G>A (p.R197H)	X	Missense		0	0.00001193	Mu et al. (2020)
c.588C>A (p.C196X)	X	Stop gain	√	0.0002177	0.00001591	Yan et al. (2020)
						Mu et al. (2020)
						Kashir et al. (2012)
						Yuan et al. (2020a)
c.570+1G>T (p.V189Cfs*12)	X	Splicing	√	NA	NA	Yan et al. (2020)
**Total in X domain**				**0.0002177**	**0.00071424**	
c.136-1G>C		Splicing		NA	NA	Zhao et al. (2023)
c.2T>C (p.M1T)		Start loss		0	0.000003981	Peng et al. (2023)
**Total**				**0.00122941**	**0.00080532**	

NA, not applicable.

Bold sections are the total of the statistics above.

Collectively, our results both expanded the pathogenic mutational spectrum of *PLCZ1* gene and emphasized the importance of *PLCZ1* gene in East Asian population.

### *PLCZ1* variations had different secondary protein structures

**FIGURE 2:**
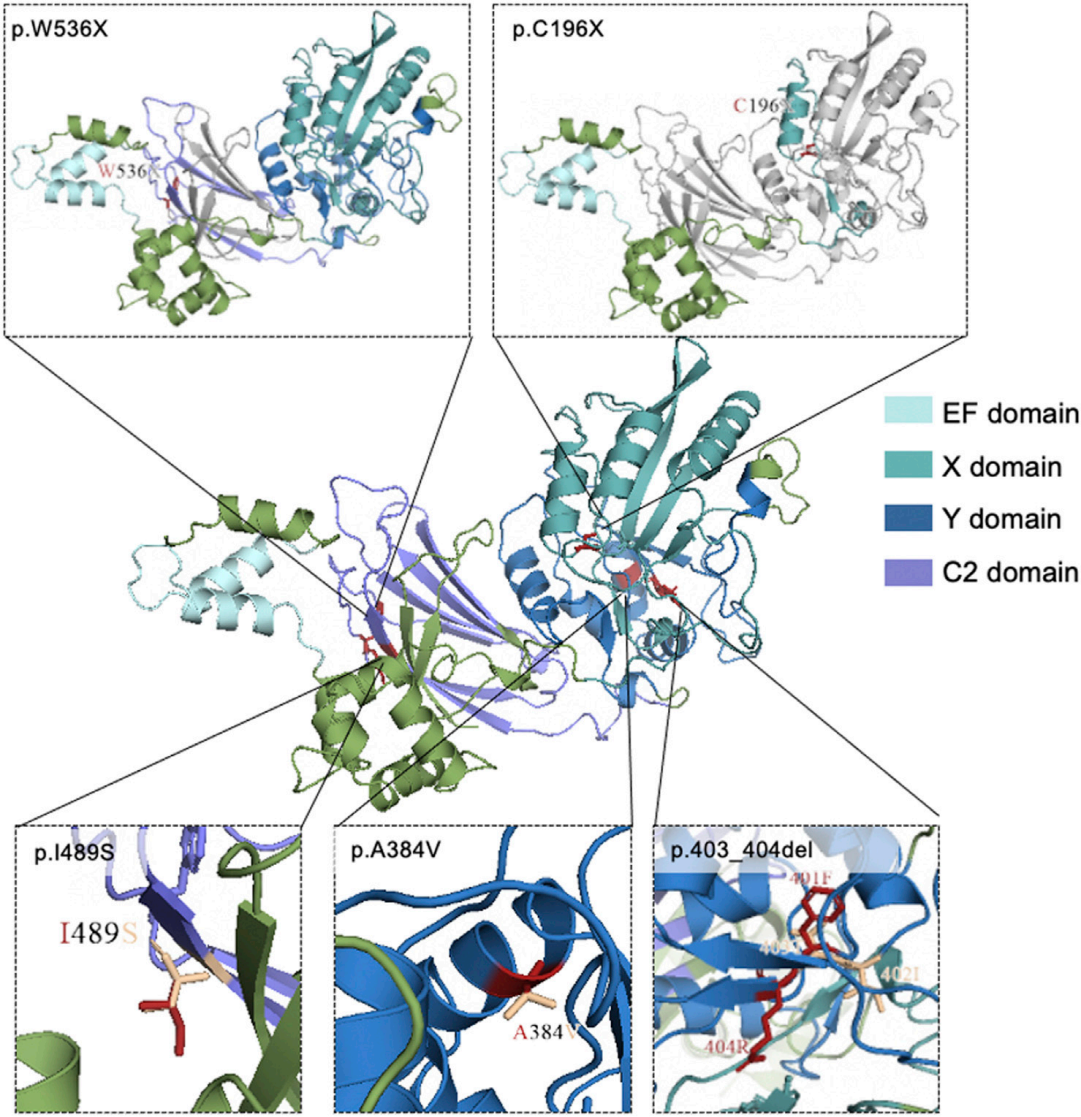
Mutational locations in *PLCZ1* proteins. 3D structure of wild-type and mutant models of *PLCZ1* protein. Wild type protein structure was shown in the center. The above pictures show the truncated peptides of p.W536X and p.C196X respectively. Grey regions indicate the lost C-terminal after the new stop codon. The red residuals in the three pictures below show the residues of missense mutations, and the wheat residuals show the residues of the wild type amino acids.

Three-dimensional models of wild-type and mutant PLCZ1 protein were mapped with PyMol software based on the predicted results of Phyre2 database (Figure 2). p.C196X and p.W536X variants produced premature termination codons. For p.A384V variant, valine substitution produced an additional side chain, which slightly increased its aliphatic property. p.I489S variant had another side chain, but the aliphatic property was decreased to a large extent.

### Novel *PLCZ1* variants were loss-of-function mutations

**FIGURE 3:**
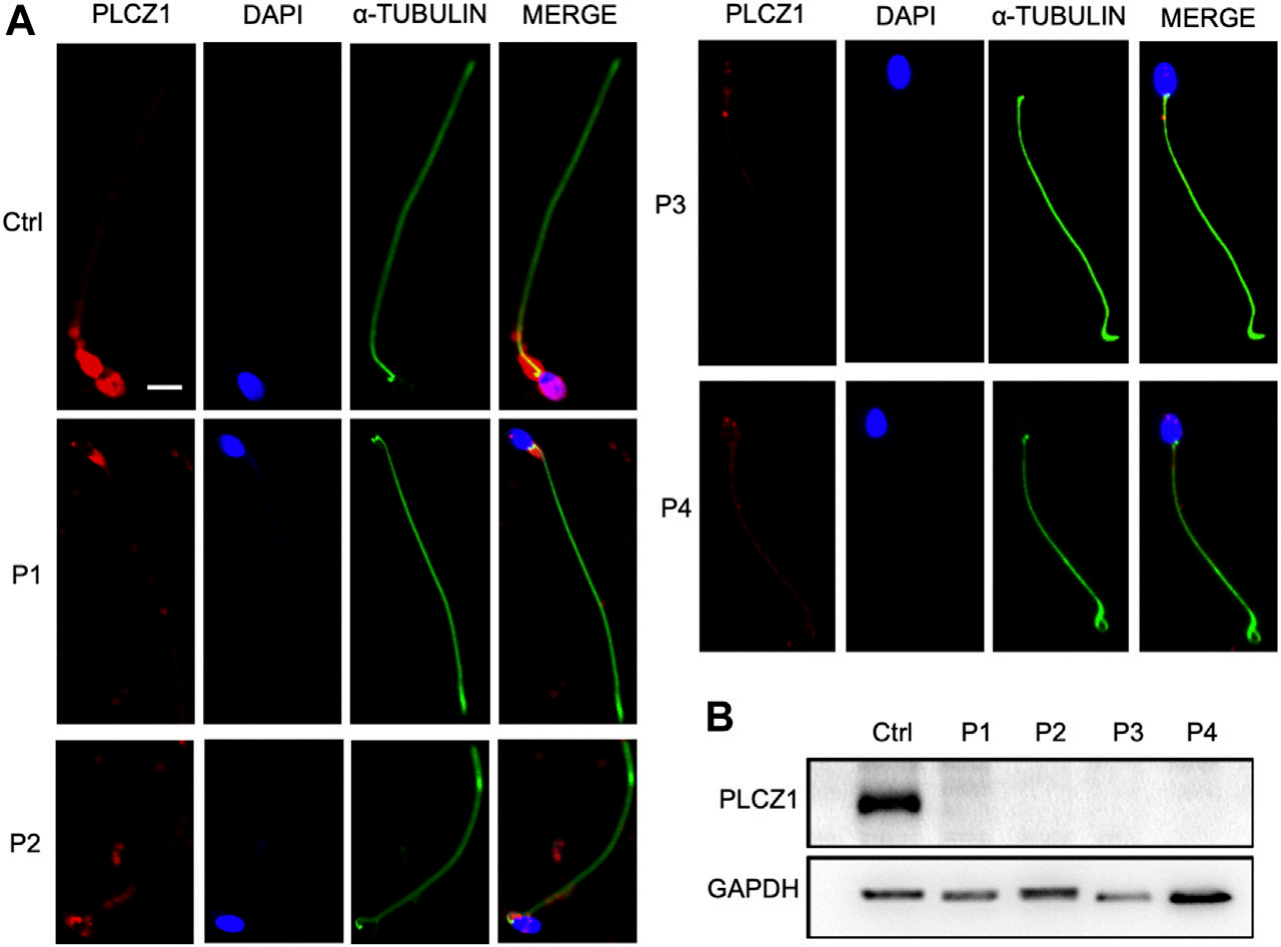
Change of protein level in the patient sperm. **(A)** Immunostaining of PLCZ1 protein by using the sperm from control and patients. DAPI is used to stain the sperm nucleus and α-TUBULIN for sperm tails. PLCZ1 is located in sperm head as in control but absent in the sperm from *PLCZ1* affected patients. **(B)** Western blot analysis of PLCZ1 level by using total protein extracted from the control and patients’ sperm. Scale bar: 5 μm.

In order to investigate the impact of these variants, distribution and expression levels of *PLCZ1* in patients’ sperms were tested. In normal sperm, PLCZ1 protein predominantly expressed in the equatorial segment. It also distributed in the acrosome, post-acrosome or a combination of these locations, which were consistent with the results shown in the previous studies (Grasa et al., 2008; Kashir et al., 2013; Yelumalai et al., 2015; Kashir et al., 2017; Agarwal et al., 2021). Contrastively, PLCZ1 protein was barely detected in sperm from the patients (Figure 3A). Western blotting analysis showed that PLCZ1 protein disappeared in all four patients as well (Figure 3B). Collectively, these results demonstrated that the *PLCZ1* variants identified in our center were loss-of-function mutations.

### Mutations in *PLCZ1* did not lead to abnormalities in sperms’ ultrastructure and semen parameters

**FIGURE 4:**
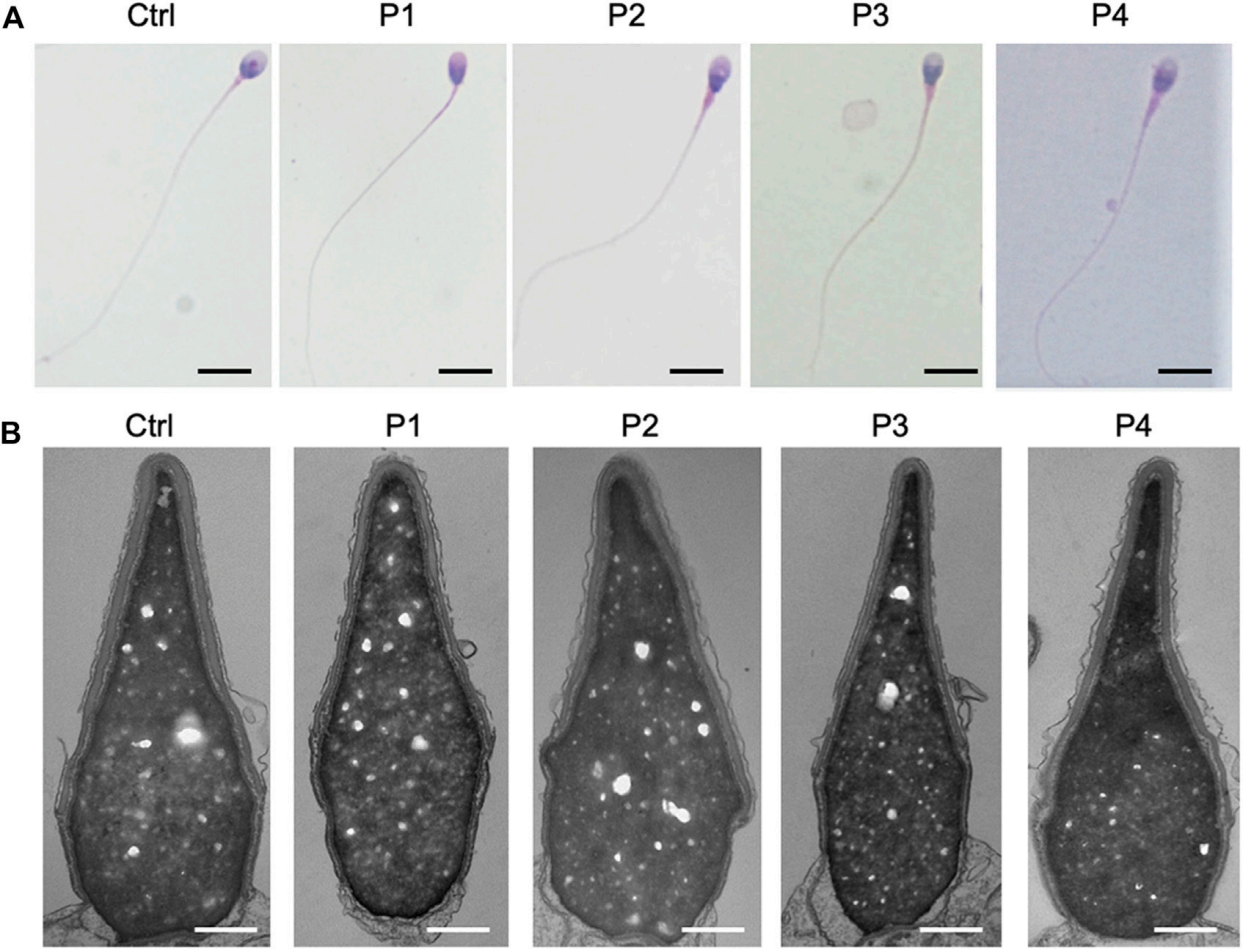
Sperm morphological analysis in *PLCZ1* affected patients. **(A,B)** H&E staining **(A)** and transmission electron micrographs **(B)** results showed no obvious malformation of sperm head and tail in *PLCZ1*-affected patients. Scale bar: **(A)** 100 μm; **(B)** 500 nm.

Sperms of the four patients in our center showed no obvious abnormalities in the semen parameters (Table 3; Supplementary Table S2). Further clinical examinations of sperm including sperm DNA fragmentation rate, acrosome reaction and seminal plasma biochemical parameters showed that these parameters mentioned above were basically normal (Supplementary Tables S2, S3). Sperms from the affected individuals manifested no morphological change under hematoxylin & eosin staining and transmission electron micrographs analysis (Figures 4A, B), which were consistent with most of the published results (Torra-Massana et al., 2019; Yan et al., 2020).

**TABLE 3:** Semen characteristics of men carrying bi-allelic *PLCZ1* variants.

Semen parameters	Patient 1	Patient 2	Patient 3	Patient 4	Reference values
Semen volume (mL)	2.0	2.2	1.8	2.0	>1.5
Sperm concentration (10^6^/mL)	53	147	94	158	>15.0
Total sperm count (10^6^)	106	323.4	169.2	316	>39.0
Motility (%)	55	59	58	76	>40.0
Progressive motility (%)	46	48	52	66	>32.0

### Polyspermy during IVF was a crucial phenotype of *PLCZ1*-related patients

Since no obvious anomalies were identified in sperm count, activity and morphology in the affected individuals, IVF was recommended for our patients in their first ART cycles. Intriguingly, during 4 IVF cycles, the oocytes were frequently polyspermic fertilized (81.8%, 18/22 oocytes) (Table 4) and no viable embryos were obtained. By contrast, the average polyspermic fertilization rate was 10.2% (1988/19423 oocytes) in our center in 2021, which was approximate to the data in other literature (van der Ven et al., 1985; Frattarelli et al., 2008). We found that the polyspermy rate in our *PLCZ1*-related patients was significantly higher than the common level (*p* < 0.0001, Fisher’s exact test). Together with the polyspermy phenotype of *Plcz1* ^*−/−*^ mice (Hachem et al., 2017; Nozawa et al., 2018) and the infertile male carrying *PLCZ1* bi-allelic mutations reported (Peng et al., 2023), we proposed that polyspermy was an important phenotype of *PLCZ1*-related infertility, suggesting the necessity of genetic test on *PLCZ1* gene for infertile males undergoing polyspermy in their previous ART cycles.

**TABLE 4:** The ART history of the four affected individuals.

Case	Cycle	No. of oocytes	No. of matured oocytes^a^	Pronucleus/pronuclei				Development outcomes of the embryos converted from 2 PN		Pregnancy outcomes
				0	1	2	≥3	No. of embryos arrested at 2-5-cell stage	No. of embryos developing to ≥6 cells	
1	IVF	10	8	1	0	0	7	0	0	—
	ICSI+AOA	18	13	7	1	4	1	2	2 (6C2, 4BC)	Abortion^b^
	ICSI+AOA	14	10	10	0	0	0	0	0	—
	ICSI-donor sperm	8	7	0	0	7	0	1	6 (7C3, 8C3, >10C3, >10C3, 4BC, 4BB)	Pregnancy
2	IVF	8	4	1	0	2	1	1	0	—
	ICSI+AOA	7	4	1	1	2	0	1	1 (6C2)	NP
3	IVF	11	10	0	0	0	10	0	0	—
	ICSI+AOA	10	NA	NA	NA	NA	NA	NA	0	—
	ICSI+AOA	7	6	4	0	2	0	0	2 (7C2, 7C3)	Live birth^c^
4	IVF	18	NA	NA	NA	6	NA	NA	0	—
	ICSI	18	13	NA	NA	6	NA	NA	0	—
	ICSI	14	NA	NA	NA	NA	NA	NA	0	—
	ICSI+AOA	16	15	2	4	7	2	6	0	—

NA, not applicable; NP, not pregnant.

^a^ The degenerated oocytes were not included here.

^b^ Chromosomal anomalies.

^c^ Boy-girl twins.

Good quality embryos: Grade 1–2, ≥7 cells.

### *PLCZ1* was a male factor leading to human early embryonic arrest

According to previous studies, AOA was a recommended intervention method for *PLCZ1*-related fertilization failure (Dai et al., 2020; Peng et al., 2023). Therefore, ICSI with AOA treatment was applied in the following cycles of the four affected couples. In total, 48 matured oocytes were gained within six AOA cycles and resulted in 15 normal zygotes with 2 PNs. 93.3% (14/15) of the 2PN zygotes were cleaved. However, 64.2% (9/14) of the cleavages were arrested at 2-5-cell stage and only one good quality blastocyst was obtained, which resulted in abortion (Table 4). During these AOA cycles, 30 matured oocytes showed 0 PN or 1 PN within 6–8 h post-fertilization. Intriguingly, we found that 53.3% (16/30) of them showed a potential to cleave. These results indicated that zygotes with 2 PN had higher potentials of cleavage than the ones with 0 or 1 PN.

In contrast, by using donated sperms, all seven matured oocytes were normally fertilized and resulted in five viable embryos. Furthermore, in our center, among 6527 IVF/ICSI cycles, 56.3% of the MII oocytes successfully developed into blastocysts after *in vitro* culture, the percentage of which was apparently much higher than that of the four affected individuals (4.2%, 2/48) (Table 5).

**TABLE 5:** Blastocyst rate in ICSI with AOA cycles of patients with bi-allelic *PLCZ1* mutations (Patient group) and total IVF/ICSI cycles in our center in year 2021 (Control group).

	Control group	Patient group
No. of blastocysts	13,625	1
No. of MII oocytes	24,205	48

In conclusion, these results suggested that *PLCZ1* was a crucial male genetic factor affecting early embryonic development and mutations in *PLCZ1* could reduce the *in vitro* developmental potential of cleavages, thus resulting in early embryonic arrest.

### Fresh cleavages transfer produced live births for patients with bi-allelic mutations in *PLCZ1*

To analyze the factors affecting the clinical outcome after ICSI and AOA treatment, we recorded the details of the treatment process of these couples. In total, we transferred five cleavages into the uteruses of the patients, including a 6C2 frozen cleavage in family 1, two fresh cleavages graded as 4C2 and 6C2 in family 2, and two fresh cleavages graded as 7C2 and 7C3 in family 3. Both 4C2 and 6C2 cleavages resulted in no pregnancy, while transfer with 7C2 and 7C3 cleavages resulted in a pair of boy-and-girl live birth twins. Considering that the cleavages with *PLCZ1* mutations had a lower *in vitro* potential to develop into blastocysts as we demonstrated above and the successful experience of live birth by fresh cleavage transfer, we supposed that fresh cleavage transfer after ICSI and AOA treatment could be a potential treatment method for male patients carrying bi-allelic mutations in *PLCZ1*, and the cell number in the cleavage may affect the clinical outcome. However, more clinical cases were needed for further verification.

### Mutational types and sites had no effects on developmental potential for embryos with mutations in *PLCZ1*

**FIGURE 5:**
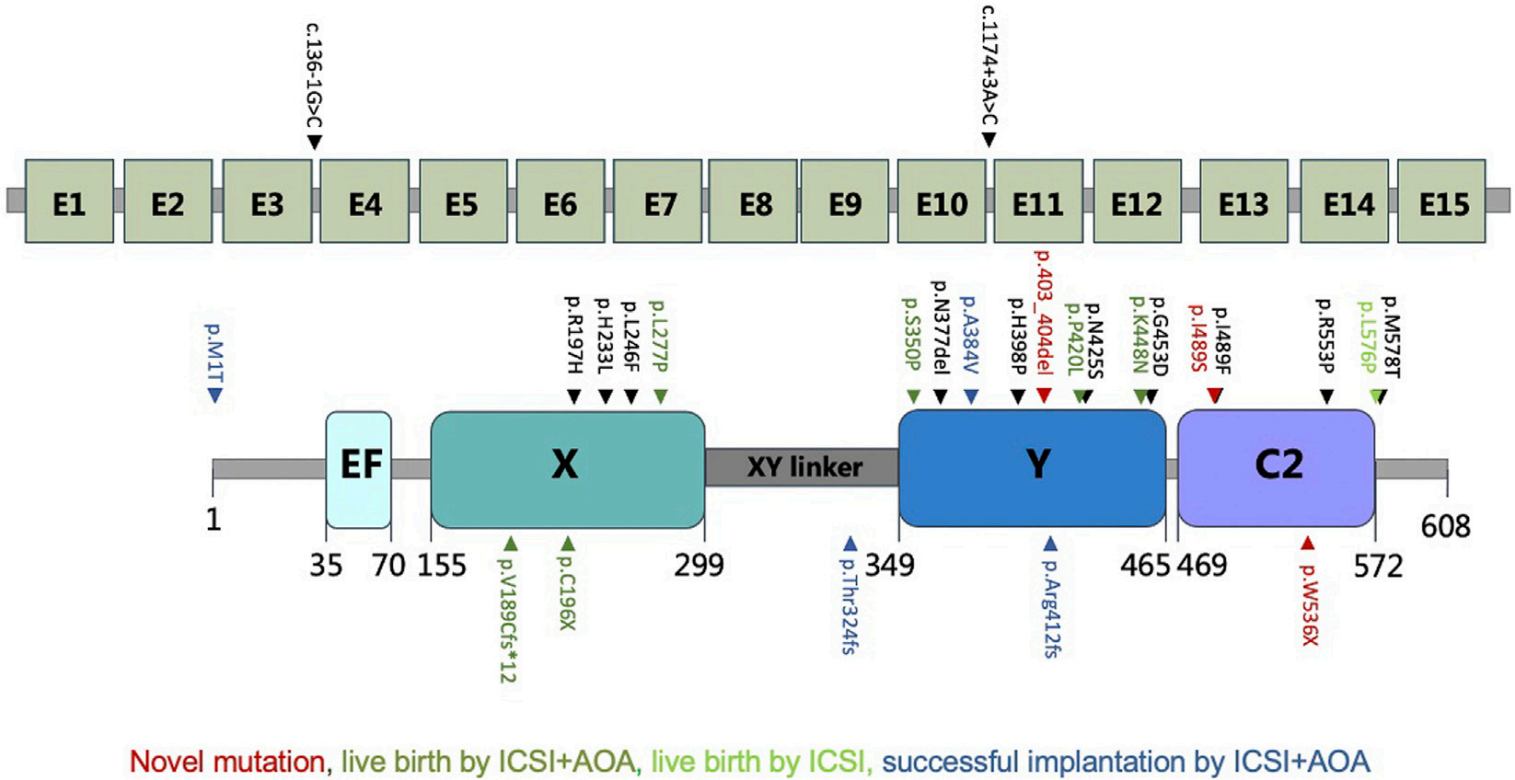
Locations of all the mutations from previous reports and our study. Red indicates novel mutations, dark green for live birth by ICSI with AOA, light green for live birth by ICSI and blue for successful implantation by ICSI with AOA.

Although AOA has been proved to be effective in rescuing fertilization failure caused by bi-allelic mutations in *PLCZ1*, only one quarter of the patients after AOA treatment gained live births. To explore whether there would be other factors affecting the outcome of AOA treatment, we studied mutational types and sites of *PLCZ1* in both our patients and *PLCZ1* bi-allelic mutational cases from the literature. We gained 26 reported mutational sites with different mutational types in 26 patients as shown in Figure 5. Among the 26 patients, seven gained successful live births. By comparing mutations in *PLCZ1* of patients with live births and without live births, we found that neither mutational types nor mutational sites of *PLCZ1* showed special effect on embryo developmental potential. Therefore, mutational types and sites were not the factors that accounted for the AOA treatment outcome. However, more *PLCZ1* bi-allelic mutational cases were needed for further analysis to figure out factors associated with AOA treatment outcome.

## Discussion

Fertilization failure is one of the most important reasons causing male infertility, which could be caused by mutations in *PLCZ1*. Twenty six male infertile individuals with homozygous or compound heterozygous *PLCZ1* variants were reported in previous studies. Here, we identified bi-allelic *PLCZ1* mutations in four infertile males, including three novel variants (c.T1466G, p.I489S), (c.1208_1213del, p.403_404del) and (c.C1607T, p.W536X), which expanded the pathogenic mutational spectrum of *PLCZ1* gene. The database showed that the carrier frequencies of the expected/known pathogenic variants in *PLCZ1* were higher in East Asian population compared with those in total population, suggesting the important role of *PLCZ1* gene in Chinese infertile male patients.

In this study, male partners from four infertile couples showed normal semen parameters, including normal sperm activity and histological shape under microscope. Therefore, IVF was recommended in their first ART cycles. However, the attempts ended up with failure. Notably, polyspermy occurred in the IVF cycles of two patients, with 81.8% (18/22) of the zygotes showing ≥3 PNs. As usual, we recommended genetic tests for the female partners, but no pathogenic variants were identified in the genes associated with female infertility. Therefore, genetic tests were suggested for the male partners, and bi-allelic mutations in *PLCZ1* gene were identified. According to previous studies, bi-allelic mutations in *PLCZ1* accounted for fertilization failure in human beings. Only one case with polyspermy was reported due to homozygous mutations in *PLCZ1* (Peng et al., 2023). In our study, polyspermy was found to be a notable phenotype in patients carrying bi-allelic mutations in *PLCZ1*. Therefore, genetic analysis would also be recommended for male patients who suffered from polyspermic fertilization even if the morphologies of their sperms were normal.

As reported in *Plcz1* ^−/−^ mouse models, polyspermy was found in eggs fertilized by *Plcz1*-null sperm after IVF, with a slight level of Ca^2+^ oscillation, suggesting that some other factors, except for PLCZ1 in the sperm head, might also play a role in triggering a slight level of calcium release (Hachem et al., 2017; Jones, 2018; Nozawa et al., 2018; Swann, 2020). This discovery corroborated the result in our study that *PLCZ1* mutations of the two affected individuals suffering from polyspermy led to total PLCZ1 protein loss.

ICSI with AOA treatment was reported to be effective in treating infertile males with mutations in *PLCZ1*. It could rescue fertilization failure to an extent and help with the achievements of successful pregnancies. In this study, we found that even with ICSI and AOA treatment, though most of the zygotes with *PLCZ1* defects could generate 2 PNs and reach cleavage stage with AOA treatment, they still had difficulties in further developing into blastocysts and arrested at 2-5-cell stages. They might be fragile and be more sensitive to the environmental damages than the normal embryos. Therefore, this study revealed that *PLCZ1* was not only crucial for fertilization, but also critical in early embryonic development.

When the female partner of patient 3 was transferred with two fresh cleavages (7C2 and 7C3), an earlier embryonic development stage than blastocyst stage, a pair of boy-girl twins were born. Thus, we suggested fresh cleavage transfer after ICSI and AOA treatment could be a potential treatment method for those with bi-allelic mutations in *PLCZ1.* Transfers with 4C2 and 6C2 cleavages failed to establish pregnancy, which suggested that the cell number was also associated with the *in vivo* developmental potential of the embryos with *PLCZ1* defects. However, more *PLCZ1*-mutational cases would still be needed to discover the factors associated with *in vitro* and *in vivo* developmental potential of embryos with *PLCZ1* defects.

We also explored other potential factors associated with clinical outcomes. We reviewed the mutational types and sites in 26 cases in terms of bi-allelic mutations in *PLCZ1*. Both severe truncating mutations, including N-terminal frameshift, stop-gain mutations, and missense mutations could result in live birth, and these mutations did not present special pattern of distribution. Therefore, we proposed that mutational sites and mutational types were not specific enough for predicting clinical outcomes of mutations in *PLCZ1*.

In conclusion, we identified novel mutations to expand the mutational spectrum of *PLCZ1* gene and suggested that people carrying bi-allelic mutations in *PLCZ1* had a high risk in polyspermy besides fertilization failure. We found that embryos from patients with bi-allelic mutations in *PLCZ1* had a lower *in vitro* developmental potential and early embryonic arrest was a new phenotype accounting for their ART failure. Therefore, fresh cleavage transfer after ICSI and AOA was applied for these patients as a potential treatment option. Our findings, together with extant knowledge of *PLCZ1* gene, might benefit the genetic counseling of infertile men in the future, and provide them with more rational ART strategies to increase the live birth rate.

## Data Availability

The datasets presented in this study can be found in online repositories. The names of the repository/repositories and accession number(s) can be found below: https://ngdc.cncb.ac.cn/gsa-human/ under HRA004448.

## References

[B1] Agarwal A. Baskaran S. Parekh N. Cho C. L. Henkel R. Vij S. (2021). 'Male infertility. Lancet 397, 319–333. 10.1016/S0140-6736(20)32667-2 33308486

[B2] Cheung S. Xie P. Parrella A. Keating D. Rosenwaks Z. Palermo G. D. (2020). 'Identification and treatment of men with phospholipase Cζ-defective spermatozoa. Fertil. Steril. 114, 535–544. 10.1016/j.fertnstert.2020.04.044 32712020

[B3] Dai J. Dai C. Guo J. Zheng W. Zhang T. Li Y. (2020). 'Novel homozygous variations in PLCZ1 lead to poor or failed fertilization characterized by abnormal localization patterns of PLCζ in sperm. Clin. Genet. 97, 347–351. 10.1111/cge.13636 31463947

[B4] Escoffier J. Lee H. C. Yassine S. Zouari R. Martinez G. Karaouzène T. (2016). 'Homozygous mutation of PLCZ1 leads to defective human oocyte activation and infertility that is not rescued by the WW-binding protein PAWP. Hum. Mol. Genet. 25, 878–891. 10.1093/hmg/ddv617 26721930 PMC4754041

[B5] Evans J. P. (2020). 'Preventing polyspermy in mammalian eggs-Contributions of the membrane block and other mechanisms. Mol. Reprod. Dev. 87, 341–349. 10.1002/mrd.23331 32219915

[B6] Frattarelli J. L. Miller K. A. Miller B. T. Elkind-Hirsch K. Scott R. T. (2008). 'Male age negatively impacts embryo development and reproductive outcome in donor oocyte assisted reproductive technology cycles. Fertil. Steril. 90, 97–103. 10.1016/j.fertnstert.2007.06.009 17765235

[B7] Grasa P. Coward K. Young C. Parrington J. (2008). 'The pattern of localization of the putative oocyte activation factor, phospholipase Czeta, in uncapacitated, capacitated, and ionophore-treated human spermatozoa. Hum. Reprod. 23, 2513–2522. 10.1093/humrep/den280 18653671

[B8] Hachem A. Godwin J. Ruas M. Lee H. C. Ferrer Buitrago M. Ardestani G. (2017). 'PLCζ is the physiological trigger of the Ca2+ oscillations that induce embryogenesis in mammals but conception can occur in its absence. Development 144, 2914–2924. 10.1242/dev.150227 28694258 PMC5592814

[B9] Jones K. T. (2018). 'Mammalian sperm contain two factors for calcium release and egg activation: Phospholipase C zeta and a cryptic activating factor. Mol. Hum. Reprod. 24, 465–468. 10.1093/molehr/gay038 30257016

[B10] Kashir J. Buntwal L. Nomikos M. Calver B. L. Stamatiadis P. Ashley P. (2017). 'Antigen unmasking enhances visualization efficacy of the oocyte activation factor, phospholipase C zeta, in mammalian sperm. Mol. Hum. Reprod. 23, 54–67. 10.1093/molehr/gaw073 27932551

[B11] Kashir J. (2020). Increasing associations between defects in phospholipase C zeta and conditions of male infertility: Not just ICSI failure? J. Assist. Reprod. Genet. 37, 1273–1293. 10.1007/s10815-020-01748-z 32285298 PMC7311621

[B12] Kashir J. Jones C. Lee H. C. Rietdorf K. Nikiforaki D. Durrans C. (2011). 'Loss of activity mutations in phospholipase C zeta (PLCζ) abolishes calcium oscillatory ability of human recombinant protein in mouse oocytes. Hum. Reprod. 26, 3372–3387. 10.1093/humrep/der336 22010140 PMC3212881

[B13] Kashir J. Jones C. Mounce G. Ramadan W. M. Lemmon B. Heindryckx B. (2013). 'Variance in total levels of phospholipase C zeta (PLC-ζ) in human sperm may limit the applicability of quantitative immunofluorescent analysis as a diagnostic indicator of oocyte activation capability. Fertil. Steril. 99, 107–117. 10.1016/j.fertnstert.2012.09.001 23040527

[B14] Kashir J. Konstantinidis M. Jones C. Lemmon B. Lee H. C. Hamer R. (2012). 'A maternally inherited autosomal point mutation in human phospholipase C zeta (PLCζ) leads to male infertility. Hum. Reprod. 27, 222–231. 10.1093/humrep/der384 22095789 PMC3241606

[B15] Kashir J. Mistry B. V. BuSaleh L. Abu-Dawas R. Nomikos M. Ajlan A. (2020). 'Phospholipase C zeta profiles are indicative of optimal sperm parameters and fertilisation success in patients undergoing fertility treatment. Andrology 8, 1143–1159. 10.1111/andr.12796 32298520

[B16] Kashir J. Nomikos M. Lai F. A. Swann K. (2014). 'Sperm-induced Ca2+ release during egg activation in mammals. Biochem. Biophys. Res. Commun. 450, 1204–1211. 10.1016/j.bbrc.2014.04.078 24769204

[B17] Mahutte N. G. Arici A. (2003). Failed fertilization: Is it predictable? Curr. Opin. Obstet. Gynecol. 15, 211–218. 10.1097/00001703-200306000-00001 12858108

[B18] Mu J. Zhang Z. Wu L. Fu J. Chen B. Yan Z. (2020). 'The identification of novel mutations in PLCZ1 responsible for human fertilization failure and a therapeutic intervention by artificial oocyte activation. Mol. Hum. Reprod. 26, 80–87. 10.1093/molehr/gaaa003 31953539

[B19] Nikiforaki D. Vanden Meerschaut F. de Roo C. Lu Y. Ferrer-Buitrago M. de Sutter P. (2016). 'Effect of two assisted oocyte activation protocols used to overcome fertilization failure on the activation potential and calcium releasing pattern. Fertil. Steril. 105, 798–806. 10.1016/j.fertnstert.2015.11.007 26632207

[B20] Nomikos M. Kashir J. Swann K. Lai F. A. (2013). 'Sperm PLCζ: From structure to Ca2+ oscillations, egg activation and therapeutic potential. FEBS Lett. 587, 3609–3616. 10.1016/j.febslet.2013.10.008 24157362

[B21] Nozawa K. Satouh Y. Fujimoto T. Oji A. Ikawa M. (2018). 'Sperm-borne phospholipase C zeta-1 ensures monospermic fertilization in mice. Sci. Rep. 8, 1315. 10.1038/s41598-018-19497-6 29358633 PMC5778054

[B22] Peng Y. Lin Y. Deng K. Shen J. Cui Y. Liu J. (2023). 'Mutations in PLCZ1 induce male infertility associated with polyspermy and fertilization failure. J. Assist. Reprod. Genet. 40, 53–64. 10.1007/s10815-022-02670-2 36529831 PMC9840742

[B23] Sanders J. R. Swann K. (2016). Molecular triggers of egg activation at fertilization in mammals. Reproduction 152, R41–R50. 10.1530/REP-16-0123 27165049

[B24] Saunders C. M. Larman M. G. Parrington J. Cox L. J. Royse J. Blayney L. M. (2002). 'PLC zeta: A sperm-specific trigger of Ca(2+) oscillations in eggs and embryo development. Development 129, 3533–3544. 10.1242/dev.129.15.3533 12117804

[B25] Swann K. Lai F. A. (2016). 'The sperm phospholipase C-ζ and Ca2+ signalling at fertilization in mammals. Biochem. Soc. Trans. 44, 267–272. 10.1042/BST20150221 26862214

[B26] Swann K. (2020). The soluble sperm factor that activates the egg: PLCzeta and beyond. Reproduction 160, V9–V11. 10.1530/REP-20-0079 32485666

[B27] Torra-Massana M. Cornet-Bartolomé D. Barragán M. Durban M. Ferrer-Vaquer A. Zambelli F. (2019). 'Novel phospholipase C zeta 1 mutations associated with fertilization failures after ICSI. Hum. Reprod. 34, 1494–1504. 10.1093/humrep/dez094 31347677

[B28] van der Ven H. H. Al-Hasani S. Diedrich K. Hamerich U. Lehmann F. Krebs D. (1985). 'Polyspermy in *in vitro* fertilization of human oocytes: Frequency and possible causes. Ann. N. Y. Acad. Sci. 442, 88–95. 10.1111/j.1749-6632.1985.tb37508.x 3860066

[B29] Wang J. Zhang J. Sun X. Lin Y. Cai L. Cui Y. (2021). 'Novel bi-allelic variants in ACTL7A are associated with male infertility and total fertilization failure. Hum. Reprod. 36, 3161–3169. 10.1093/humrep/deab228 34727571

[B30] Wang T. Cao B. Cai Y. Chen S. Wang B. Yuan Y. (2022). 'Plcz1 deficiency decreased fertility in male mice which is associated with sperm quality decline and abnormal cytoskeleton in epididymis. Int. J. Mol. Sci. 24, 314. 10.3390/ijms24010314 36613757 PMC9820195

[B31] Xin A. Qu R. Chen G. Zhang L. Chen J. Tao C. (2020). Disruption in ACTL7A causes acrosomal ultrastructural defects in human and mouse sperm as a novel male factor inducing early embryonic arrest. Sci. Adv. 6, eaaz4796. 10.1126/sciadv.aaz4796 32923619 PMC7455188

[B32] Yan Z. Fan Y. Wang F. Yan Z. Li M. Ouyang J. (2020). 'Novel mutations in PLCZ1 cause male infertility due to fertilization failure or poor fertilization. Hum. Reprod. 35, 472–481. 10.1093/humrep/dez282 32048714

[B33] Yelumalai S. Yeste M. Jones C. Amdani S. N. Kashir J. Mounce G. (2015). 'Total levels, localization patterns, and proportions of sperm exhibiting phospholipase C zeta are significantly correlated with fertilization rates after intracytoplasmic sperm injection. Fertil. Steril. 104, 561–568. 10.1016/j.fertnstert.2015.05.018 26054556

[B34] Yeste M. Jones C. Amdani S. N. Patel S. Coward K. (2016). Oocyte activation deficiency: A role for an oocyte contribution? Hum. Reprod. Update 22, 23–47. 10.1093/humupd/dmv040 26346057

[B35] Yuan P. Yang C. Ren Y. Yan J. Nie Y. Yan L. (2020a). 'A novel homozygous mutation of phospholipase C zeta leading to defective human oocyte activation and fertilization failure. Hum. Reprod. 35, 977–985. 10.1093/humrep/dez293 32142120

[B36] Yuan P. Zheng L. Liang H. Lin Q. Ou S. Zhu Y. (2020b). 'Novel mutations in the PLCZ1 gene associated with human low or failed fertilization. Mol. Genet. Genomic Med. 8, e1470. 10.1002/mgg3.1470 32840018 PMC7549595

[B37] Zhao S. Cui Y. Guo S. Liu B. Bian Y. Zhao S. (2023). 'Novel variants in ACTL7A and PLCZ1 are associated with male infertility and total fertilization failure. Clin. Genet. 103, 603–608. 10.1111/cge.14293 36593593

