# Supplementary material for: Novel mutations in PLCZ1 lead to early embryonic arrest as a male factor
Source: Front Cell Dev Biol. 2023 May 16;11:1193248. doi: 10.3389/fcell.2023.1193248 (PMC10227596; doi:10.3389/fcell.2023.1193248)
Supplement: Supplementary file 1 [file DataSheet1.pdf]

Supplementary Materials

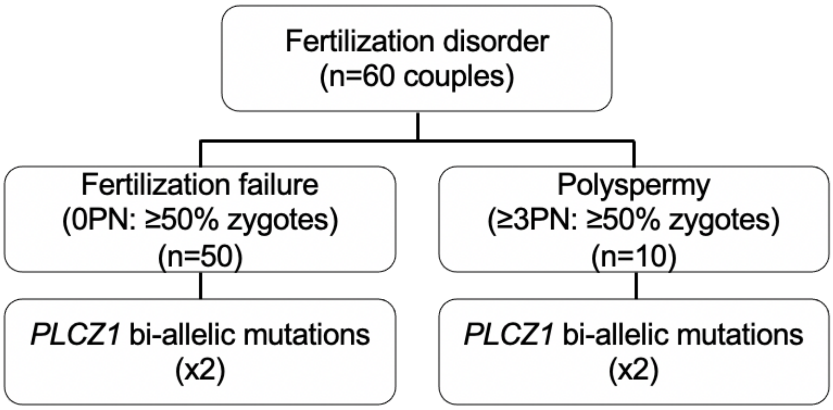

Figure S1. Cohort in this study and contribution of *PLCZ1* variants.

Table S1. Genomic PCR primers used to amplify *PLCZ1* mutations for Sanger sequencing.

Sites		Primer sequences (5′ to 3′)	Product Size (bp)
M1	F	ACCATGAATGCATACTTGTGT	242
	R	ATGGACTCATTTATGCATTTA	
M2	F	ATTACACGAGTCTGCTGCTTC	210
	R	GGAGCTGACTTAGAGTCCATC	
M3	F	GCTTCCAGGACAAAGTTCAA	622
	R	TCAAATGGTGTTTGAGGAGA	
M4	F	TCTCAAACTTTCCTGGCCAT	756
	R	TTGGGAGTAGGGATGTTTCA	
M5	F	GGCTTCAACCCAGATAGGAAT	697
	R	TGCCCTACTGTGAAAATCAA	
M6	F	GCTAAAGAATTGCCTAAGTACATC	886
	R	CTGGTTGCCACCCATCATCA	

bp, base pairs; F: forward primer; R: reverse primer.

Table S2. Semen characteristics of men carrying bi-allelic *PLCZ1* variants.

Semen Parameters	Patient 1	Patient 2	Patient 3	Patient 4	Reference Values
semen liquefaction time (min)	20	20	20	20	≤60
Total motile sperm count (10^6)	58.3	190.8	98.1	240.2	
Non-progressive motility (C, %)	9	11	6	10	0-60
Progressive motile sperm count (10^6)	48.8	155.2	88	208.6	
Immotile sperm (D) (%)	45	41	42	24	0-60
Round cell (10^6/ml)	0.2	0.2	0.2	1.3	0.1-0.9
Leukocyte (10^6/ml)	0.1	NA	0.1	0.3	0-0.9
Survival rate (eosin) (%)	79	NA	94	95	58-100
MAR adsorption rate (%)	5	NA	6	6	0-10
Fructose test	NA	NA	positive	positive	positive
Sperm DNA fragmentation rate (%)	20.24	13.82	14.53	8.29	≤15
Acrosome reaction (%)	NA	NA	2	5	0-10
Morphologically normal sperm (%)	6	5	6	5	
Abnormal head (%)	61	69	67	69	
Abnormal midpiece (%)	0	1	1	2	
Abnormal Flagella (%)	1	2	1	1	
Mixd malformation (%)	32	23	25	23	

NA, not available

*Mean Value of 3-4 independent tests

Table S3. Seminal plasma biochemical parameters of men carrying bi-allelic *PLCZ1* variants.

Seminal plasma biochemical parameters	Patient 3	Patient 4	Reference Values
Zn (mmol/L)	3.56	3.4	1.09-4.86
TAG (U/L)	365.87	477.69	109.63-570.76
NAG (U/L)	29.04	41.14	>10.12
FRU (mmol/L)	24.49	9.49	>6.04
ACP (U/ml)	724.74	846.28	152-1665

*Mean Value of 3-4 independent tests
